# Nova Scotia’s Deemed Consent for Deceased Organ Donation: Family Member Perspectives and Experiences in the ICU Setting

**DOI:** 10.1097/TXD.0000000000001713

**Published:** 2024-10-10

**Authors:** Aimee J. Sarti, Stephanie Sutherland, Matthew J. Weiss, Alain Landry, Heather Hemming, Jade Dirk, Ken Lotherington, Stephen Beed

**Affiliations:** 1 Department of Critical Care, Ottawa Hospital, Ottawa, ON, Canada.; 2 Ottawa Hospital Research Institute, Ottawa, ON, Canada.; 3 Population Health and Optimal Health Practices Research Unit, Trauma-Emergency-Critical Care Medicine, CHU de Quebec-Université Laval, Quebec City, QC, Canada.; 4 Legacy of Life Organ Donation Program, Nova Scotia Health, NS, Canada; 5 Canadian Blood Services, Ottawa, ON, Canada.; 6 Department of Critical Care, Dalhousie University Faculty of Medicine, Halifax, NS, Canada.

## Abstract

**Background.:**

The purpose of this study was to explore the experience of family members of potential organ donors in the intensive care unit following the change to deemed consent legislation in Nova Scotia.

**Methods.:**

This was a qualitative study with semistructured, in-depth interviews with 17 family members who were asked to make an organ donation decision on behalf of patients admitted to the intensive care unit in Nova Scotia. We analyzed themes using a descriptive approach. Participants were recruited from the organ donation organization in Nova Scotia, Canada.

**Results.:**

Participant awareness and knowledge of the Human Organ and Tissue Donation Act legislation varied from individuals having no awareness and knowledge of the bill to those who had awareness and optimism that the legislation would be beneficial for increasing organ donation rates in the province. Other themes emerging from the interviews included (1) COVID context, (2) quality of healthcare professional care, (3) family support, and (4) barriers to donation (waiting, consent questionnaire, and patient transfers).

**Conclusions.:**

The Human Organ and Tissue Donation Act legislation included enhanced support, which was viewed positively by family members. There is a need for continued evaluation as most participants felt it was too early to see the tangible impacts of the newly implemented legislation.

There continues to be a global shortage of transplantable organs within Canada. One of the most frequently suggested solutions to narrow this gap is the introduction of deemed consent legislation, also known as an opt-out system. Specifically, deemed consent means, in the absence of a formal objection, the individual is taken as having consented to donation.^1^ Many countries already have a deemed consent model in place, including Austria, Belgium, Brazil, Croatia, Wales, England, and Spain, albeit with mixed effects.^2^

On January 18, 2021, with the passage of the updated Human Organ and Tissue Donation Act (HOTDA), Nova Scotia, a small and geographically dispersed province in Canada with one tertiary hospital, became the first jurisdiction in North America to adopt a deemed consent model for deceased organ and tissue donation. This law marked a significant change in how consent for deceased organ donation is considered in intensive care units (ICUs). Most importantly, the baseline position moved from a presumption of neutrality to a presumption of yes to donate, consistent with widespread support for organ and tissue donation within the province.^3^ In practice, there are 2 different approaches to deemed consent: soft opt-out and hard opt-out. The former involves the family in decisions, whereas the latter strictly permits the recovery of organs in the absence of a formal objection.^2^ In Nova Scotia, the HOTDA is a soft deemed consent because substitute decision makers (as defined by HOTDA) can give or withhold their consent for donation on behalf of the deceased, even if the person had registered an intent to donate.^4,5^

Understanding that the literature suggests consent model changes without comprehensive system reform would likely not impact donation and transplant rates, the provincial government provided support for donation-focused physicians, increased the number of organ and tissue donation coordinators throughout the province, supported healthcare professional education, added a family support liaison role, created opportunities for professional development, and launched public awareness campaigns.^3^ These well-described components of high-performing donation systems were implemented with the intent to increase donation and transplantation rates in the province. Given the recent implementation of deemed consent in Nova Scotia, it is important to evaluate the impacts of the legislative change and the transition on family members and their experiences with organ donation to inform future program development and determine any potential impacts of this change. To oversee evaluation activities, the Legislative Evaluation: Assessment of Deceased Donation Reform Program was created to evaluate the implementation processes and overall impact of HOTDA and the health system transformation in Nova Scotia.^6^ This study is a qualitative subcomponent of the overall evaluation. Our aim is to describe the experiences of family members of potential organ donors in the ICU following the change in legislation to a deemed consent model.

## MATERIALS AND METHODS

### Study Design

This was a qualitative descriptive study involving in-depth interviews with family members, following the implementation of HOTDA legislation, who were invited to consider organ donation after: (1) death determination by neurologic criteria or (2) a decision to withdraw life-sustaining measures and death determination by circulatory criteria. This study is an extension of our previous work on family-centered experiences with bereaved families.^7-10^ All study recruitment, data collection, data analysis, and reporting followed the Consolidated Criteria for Reporting Qualitative Research checklist requirements (see **Table S1, SDC,** http://links.lww.com/TXD/A704). This study received ethics approval from the Nova Scotia Health Research Ethics Board.

### Recruitment

The Legacy of Life Organ Donation Program in Nova Scotia compiled a database of eligible family members, including their names, telephone numbers, addresses, sex, relationships with the patient, the donation decision, and the manner of death determination (neurologic or circulatory criteria). Following a period of 2 mo after the patient’s death, organ donation organization staff contacted family members to explain the purpose of the study and to request their permission to be contacted by research staff. Next, the second author (S.S.) contacted family members who agreed with the research to further explain the study and schedule a telephone interview with those who consented.

### Data Collection

Informed by earlier studies of family member experiences, we developed a semistructured interview guide.^8-10^ An interdisciplinary team of investigators with experience in critical care, palliative care, organ donation, medical education, and sociological and qualitative research methods provided feedback on the guide.^7^ The semistructured interview guide began by asking family members how they first learned of their loved one’s traumatic event and proceeded asking them to describe the end-of-life process, the organ donation experience, and finally, their post-hospital bereavement. In collaboration with the Nova Scotia Organ Donation Organization, additional questions were included to explore awareness of legislative change in the province and potential impacts on their experience. The guide was semistructured in nature to provide structure and permit the exploration of new themes. Telephone interviews were conducted by an experienced qualitative researcher (S.S.). Telephone interviews took place from January 2022 to May 2023 and lasted from 45 to 90 min in duration.

### Data Analysis

All interviews were audio-recorded, transcribed verbatim, and uploaded into ATLAS.ti, a qualitative research software program to facilitate data management and analysis (Atlas.ti software, Scientific Software Development GmbH, Berlin, Germany). We followed a qualitative descriptive approach to data analysis, including deductive coding the data from interviews and engaging in thematic analysis whereby themes were generated directly from the data.^11-13^ In particular, each transcript was coded separately by 2 researchers (S.S. and A.J.S.), who then met to discuss the codes and their definitions, and to resolve any disagreements.^13^ The codebook was iteratively developed.

## RESULTS

Overall, 17 family members consented and participated in 16 semistructured interviews (Table 1). Interviews were conducted with one family member except in one case, whereby a mother and father were interviewed together. Most of the interviewees were female (N = 13). Relationship of participants with patients was predominantly a spouse (N = 6), followed by mothers (N = 4), children (N = 3), and siblings (N = 3).

**TABLE 1.** Participating FM characteristics as related to patients

FM characteristic	Total (N = 17)^*a*^
FM sex	
Female	13
Male	4
FM relationship with patient	
Mother	4
Father	1
Spouse	6
Child	3
Sibling	3

^*a*^Seventeen participating family members for 16 patients (one interview was with mother and father).

FM, family member.

Potential organ donors (N = 16) in our study included 9 men and 7 women (Table 2). The average age of potential donors was 50 y, including both pediatric and adult individuals. There were 8 patients for whom death was declared by cardiocirculatory criteria, 6 by neurologic criteria, and 1 patient who had signed their donor card but the family ultimately declined donation, and 1 other patient who did not have any prior consent registered whose family declined donation as they did not want their loved one transferred out of their community to Halifax for the donation surgery. It should be noted that the Nova Scotia Legacy of Life donation organization does not record the cause of death for individuals who do not proceed to a donation attempt. The most common cause of death was a cerebrovascular accident.

**TABLE 2.** Characteristics and causes of death of potential donors

Patient characteristics	Total (N = 16)
Mean age, y	50
Sex	
Female	7
Male	9
Patient transfer	
No. of patients transferred to tertiary hospital	11
Cause of death	
Cerebrovascular accident	7
Traumatic brain injury/cerebral hemorrhage	3
Hypoxic/ischemic brain injury	2
Other	2
Death determination	
DNC	5
DCC	9
Unknown^*a*^	2
Registered donor status	
Registered donor 1^*b*^	9
Registered donor 2^*c*^	5
Family overrule patient donor status (registered donor 1)	1
Family decline	1

^*a*^Organ donation organization does not record patient cause of death if donation is declined.

^*b*^Registered donor 1: consent to donate all organs and tissues.

^*c*^Registered donor 2: consent to donate some organs and tissues.

DCC, determination by circulatory criteria; DNC, determination by neurologic criteria.

**FIGURE 1.**
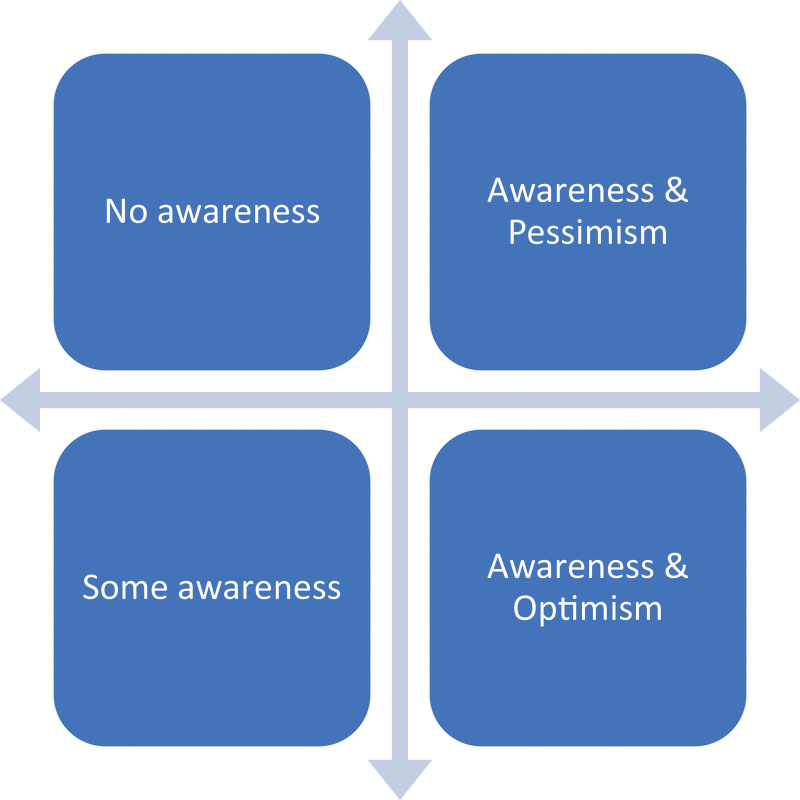
Participants awareness of the Human Organ and Tissue Donation Act (HOTDA).

We asked all participants whether they were aware of the new legislation (HOTDA) during their time in the hospital and, if so, whether it had any impact on their decision (or not) to donate their loved one’s organs. As illustrated in Figure 1, there was a range of participant responses; some family members had no awareness, there was a group with some awareness, another group indicated they were aware of the new legislation but were pessimistic about its impact, and a final group that was aware and felt optimism regarding potential impact of HOTDA. Family member responses were almost equally distributed among the 4 response domains (see **Tables S1A and S1B, SDC,** http://links.lww.com/TXD/A704 for a complete data display of participant quotes).

### No Awareness

Some family members were not aware of the new legislation at all. Aside from not knowing about the legislation, one family member stated that the topic of deemed consent did not come up during their ICU stay.

Interviewer: Did any conversations come up while you were in the hospital regarding the new deemed consent legislation in Nova Scotia?

Participant: What?

Interviewer: Last January Nova Scotia passed legislation whereby a person no longer needs to indicate they are a donor….

Participant: No nothing like that….

### Some Awareness

Other family members explained that they were not certain about how the legislation worked, but they did recall having heard about it before their time in the ICU.

I don’t know exactly how it [new legislation] works but I think it has something to do with if there’s a no on your card, okay. If there’s not a yes on your card and there’s not a no, they assume they can harvest organs. That’s the impression I get. I don’t know much about it. Now I would think they would have to ask anyway. I can’t see them being able to do it without asking.

### Awareness and Pessimism

Our findings revealed a group of family members who were familiar with the legislative changes to organ donation consent but felt the legislation was not effective because family members were still consulted for donation decisions, thus diminishing the potential impact of the legislation.

[The legislation] is useless. A useless law or regulation whatever you want to call it. It’s kind of like straw legs. There is not much to it. Even though I signed my donor card, once I went to the hospital, they would see that I want my organs to be donated but then they’d ask my kids and if that’s what I wanted they probably would follow it but who knows so you really need to focus on the people who come in control when the person dies. You want them on the side of donation.

### Awareness and Optimism

In contrast, a group of family members were aware of the legislation and felt as though it would be beneficial in increasing organ donation rates in the province.

We have big hopes for it, I mean we’re creating new positions and new social work positions. I think we have big hopes. I mean, more available organs and in a more lively transplant program and a shorter wait list, that’s what we hope. I don’t think we’ve seen any action yet though.

In addition to family member perspectives regarding the degree to which they were aware of the new legislation, additional themes from the interviews included COVID context, quality of healthcare professional care, family support, barriers to donation (waiting, consent questionnaire, and medical transfers), and awareness/knowledge of deemed consent legislation.

### COVID-19 Context

Family ICU stays took place during the pandemic, and as such, COVID concerns overshadowed hospital experiences. Family members expressed gratitude and recognition of the work done by the care team to care for their loved ones and to ensure the opportunity for donation.

We brought up donation and were surprised. It was like they were “woah we’re not there yet”, but they also panicked because they weren’t doing transplants, they shut that all down and the teams had been redistributed. We found out later if I’m remembered the details correctly, is that our request at that moment caused them to have some official conversations amongst themselves to pull together a team which had been on hold because of COVID, and they started doing some work as a group to pull together some surgeons and some stuff to make it happen. Because of COVID it took a week to organize. We were given the opportunity to sit with him for a week, even though we knew he was not going to get better. So, we were able to be at the hospital with him.

### Quality of Healthcare Professional Care

Overall satisfaction with the care provided in the ICU was high. Participants praised the staff in the hospital for their compassionate care.

The nurses were phenomenal. They were giving care as if he was still alive. Even when they were taking blood and doing whatever they were like, “here’s a little poke [pt. name].” They treated him like he was still a living child.

The staff in the hospital were so good to me and my dad, I can never say enough good things. The day Dad passed the doctor came in and offered me his phone and asked me if I wanted to use it to play some music for my father. And, of course, I played his group. That’s how caring they were. Like, the doctor, gave me his phone for two hours.

### Family Support

Family members described feeling supported while in the hospital, particularly at key moments during end-of-life care and donation processes of care.

There were two other women that I met initially from the donor program. And who did the intake paperwork and stuff like that. Then [a different person from Legacy of Life] came, I think on Tuesday. And she said there are different things that people do. She gave me some examples of things that they have done, and that was good as I had never really heard of that. She had the necklaces…. There are two necklaces, and one is a small heart, and one is a larger heart…. So that was my way to say goodbye.

It was one of the ladies from the Legacy of Life … she stayed with us and walked us out to our vehicle.

### Barriers

Barriers included time spent waiting while in the hospital for news of a loved one’s condition, the extra time required for donation to occur, the medical questionnaire, and transfers from community hospitals to the one tertiary hospital that performed the donation.

#### Extra Time

Some family members described the additional time required for donation as particularly difficult. In particular, the uncertainty as to why they were waiting was, at times, unbearable.

Once we got to Halifax, the people were good, but you were only allowed 3 in the hospital because of COVID so they gave us a family room. It was a room with no windows. It was like 10 x 10 with a couch. The worst part about it was that it took 3 days. So, that is a long period, three days, before they decided what they were going to take. Now I know they need to line up the others who need the organs but 3 days? Sitting around for three days waiting for them to make up their minds, which I guess is how it works but don’t leave everybody sitting there. I am going to change my donor card to no. I just can’t put my family through that.

We told them that we can’t wait forever. Like I can’t wait for more than 3 days. We just couldn’t wait any longer to get themselves organized. My husband stayed while the nursing staff prepped him for surgery, then we left. An older nurse told me that she’d take care of him like her grandchildren … she promised.

#### Patient Transfers

As previously stated, Nova Scotia is a small yet geographically dispersed province with 1 tertiary center that performs organ donation. Given this, most patients in the study needed to be transferred from smaller centers to the tertiary hospital in Halifax.

They tried to get what they call Life Flight here which is the same as an air ambulance. And they could land in [city] right beside the hospital, but they weren’t flying because it was too windy. And they have a fixed wing which they can land close to [city], and it wasn’t flying either, so they said, ‘ok we’re going to have to go by road which is a two-hour ride to Halifax.

#### Medical Questionnaire

Some family members were not well prepared for the required medical questionnaire as part of the donation process. Participants described the questions as very personal and hard to answer, given their state of mind.

I went through the [profanity] roof, I jumped over the table at the woman who started asking me about [pt.’s name] and sexually transmitted diseases, I swear I could have [profanity] ripped off her face. I mean like he is [pt.’s age, pediatric] [profanity]; he hasn’t been sexually abused. [profanity] off. Like gonorrhea. Like, [profanity], I lost it.

## DISCUSSION

In this study, we described the experiences of family members of potential organ donors in the ICU following the change in the donation legislation of Nova Scotia to a deemed consent model. A range of perspectives were found regarding participant awareness of the legislation. It should be noted that the revised HOTDA came into effect in January 2021, nearly 1 y into the global COVID-19 pandemic. This created challenges to undertaking extensive clinician training, given the additional strain on the health system, particularly emergency departments and ICUs. Additionally, pandemic-related restrictions meant a planned, face-to-face educational rollout was not possible. Additionally, the low population density of Nova Scotia made broad delivery of education a challenge, particularly because low-volume healthcare centers have limited donation opportunities.^14,15^ Interview data from our participants were divided into 4 groups: no awareness, some awareness, awareness and pessimism, and awareness and optimism. A lack of awareness of the HOTDA legislation did not have a negative impact on family members’ experience in the hospital, as all participants described being well supported by compassionate ICU staff. Participants in the awareness and pessimistic group supported donation; however, they felt the legislation was too soft and would not produce the desired impact. In contrast, family members in the awareness and optimistic group felt that HOTDA would improve future donation rates in Nova Scotia. Earlier studies of HOTDA in Nova Scotia have found similar results: individuals mixed in their awareness and understanding.^16^ Together, these findings are consistent with international reports that suggest that the consent model alone is insufficient to substantially improve donation and transplantation rates without adequate education and human resource support.^17,18^

Changing donation policies is a step that must be taken with the family in mind. That is, it is imperative to build trust with families and provide family support and high-quality care for potential donors. The efficiency of any donation consent model relates to several important considerations, such as accommodating next-of-kin and educating the public on donation and transplantation measures.^19^ Our prior work found gaps in the way families are supported during end-of-life and donation care, particularly regarding a perceived lack of support during key moments in the donation process.^8-10^ With the implementation of HOTDA, the government of Nova Scotia collaborated with the Legacy of Life Organ Donation Program to provide additional support and education for the donation system, including hiring and training more coordinators, creating a family support liaison role/social worker position and supporting donation-focused physician.^16^ The United Kingdom has identified system improvements with the addition of family support roles.^17,20^ Family members in the current study felt well supported and viewed the donation experience positively. Investments in infrastructure, specifically family support roles, appear promising to the overall donation experience.

Family involvement in consent legislation is critically important for a high-functioning donation system.^21^ Costa-Font et al^22^ documented that family consent legislation plays a central role in influencing both organ donation intentions and actual donation rates. Specifically, they found that family consent reduces the influence of regulatory policies on actual donation. Findings from other countries suggest strong evidence that the importance of the legislative environment is moderated by the role of the family in vetoing donations.^23^ These findings demonstrate the need to focus on strategies to encourage people to talk about organ donation with their families and friends.^1^ Evidence suggests that families who are aware of their loved one’s donation decisions are less likely to veto donation.^22,24,25^

Families in this study identified barriers in the donation process, including the extra time that donation requires, the unique geography of Nova Scotia, requires patients to be transferred to the one tertiary hospital for donation, and families’ general uncomfortableness while the medical questionnaire that was conducted on behalf of their loved one. Although the issue of time is not new and has appeared in prior studies,^8-10^ the family unease with the medical questionnaire has not been widely discussed in the donation literature. Perhaps it is time to reexamine the necessity of the medical questionnaire, as one could argue that appropriate laboratory and medical record reviews are likely going to reveal the necessary information. Families need frequent and ongoing communication from the healthcare team as to why they are waiting and to be provided explanations as to why certain pieces of information are required for potential donation and subsequent transplantation.

### Limitations

Our study has several limitations. There may be selection bias in terms of who agreed to an interview, which could be impacted by family members who consented to donation, those who had fairly good experiences and trust in research, or those who had very bad experiences and wished to express it. Our relatively small number of participants limits broad generalizations; however, this study adds to the current dearth of literature regarding family involvement in deemed consent legislative changes. Finally, interviews were conducted with English-speaking Canadian family members only, and as a result, other ethnicity-based views were not included.

## CONCLUSIONS

In this study, we found family members experienced high levels of overall satisfaction with the care and support they received in the tertiary hospital. Based on the findings in our prior research, we suspect the increase in resources, including increases in the number of donor coordinators as well as enhanced donor coordinator training/education, contributed to families feeling well supported during their donation journey. We found a range of perspectives regarding participant awareness of the HOTDA legislation, including no awareness, awareness and pessimism, some awareness, and awareness and optimism. Undoubtedly, it is too early to determine the impacts of the Nova Scotia deemed consent legislation, and ongoing research and evaluation are necessary. A spotlight is shining on what is happening in Nova Scotia as other Canadian provinces are discussing enacting similar legislations while the global debate around deemed consent as a solution to organ shortages ensues.

## ACKNOWLEDGMENTS

The authors extend their sincerest appreciation to all the family members who took the time to share their thoughts and experiences.

## Supplementary Material


