# Supplementary material for: Nova Scotia’s Deemed Consent for Deceased Organ Donation: Family Member Perspectives and Experiences in the ICU Setting
Source: Transplant Direct. 2024 Oct 10;10(11):e1713. doi: 10.1097/TXD.0000000000001713 (PMC11469852; doi:10.1097/TXD.0000000000001713)
Supplement: Supplementary file 1 [file txd-10-e1713-s001.pdf]

Table S1: Domains and items covered by the COREQ checklist

Domain	Total # of Items	Details of Items	Page location in manuscript
Domain 1: Research team and reflexivity	8 items	Personal characteristics (5 items)<br>• Interviewer, credentials, occupation, gender, experience & training<br>Relationship with participants (3 items)<br>• Prior relationship, participant knowledge of interviewer, interviewer characteristics	• Data collection (p 6)<br>• N/A* (no prior relationship – see recruitment details - recruitment(p 5-6)
Domain 2: Study design	15 items	Theoretical framework (1 item) Participant selection (4 items)<br>• Sampling, method of approach, sample size and non participation<br>Setting (3 items)<br>• Setting of data collection<br>• Presence of non participants<br>• Description of sample<br>Data collection (7 items)<br>• Interview guide<br>• Repeat interviews<br>• Audio/visual recording<br>• Field notes<br>• Duration<br>• Data saturation<br>• Transcripts returned	• Data collection - see development of guide (p 6)<br>• Design & setting, recruitment (p 5-6)<br>• Design & setting (p 5)<br>• N/A<br>• Design & setting, recruitment (p 5-6)<br>• Data collection (p 6)<br>• N/A<br>• Data analysis (p 6)<br>• N/A<br>• Data collection (p 6)<br>• All interviews were coded<br>• Upon request
Domain 3: Analysis and findings	9 items	Data analysis (5 items)<br>• Number of coders<br>• Description of coding tree<br>• Derivation of themes<br>• Software	• Data analysis (p 6)<br>• Data analysis (p 6)<br>• Data analysis (p 6)<br>• Data analysis (p 6)<br>• Data analysis (p 6)

		• Participant checking<br>Reporting<br>• Quotations presented<br>• Data and findings consistent<br>• Clarity of major themes<br>• Clarity of minor themes	 <br>• Results (p 6-9)<br>• Results (p 6-9)<br>• Results (p 6-9)<br>• N/A

*N/A is not applicable

Supplement 1a Themes: Participant awareness of HOTDA legislation

No awareness

NS_016

R: *Did any conversations come up while you were in the hospital regarding the new deemed consent legislation in Nova Scotia?*

I: *The legislation being? I'm not familiar with that.*

NS_015

I don't know what that is.

Awareness & Pessimism

NS_006

It doesn't really seem to have made an impact. There are still wait lists for transplants. Even though the legislation has passed, people are still being given a choice and families are still being asked the question. I just don't think it's made a difference.

Some awareness

NS_025

Deemed consent wouldn't have been a factor in our decision because we had all signed our donor cards. It was a non-issue for us.

NS_003

It will be interesting to see how this consent thing works would in Nova Scotia. There was an article just last week about it. Just something on the news, if you want to Google search it somehow, they were just talking about its kind of too early to tell with COVID and everything how things have played out, if

Awareness & Optimism

NS 013

Yes, I did know about the deemed consent. I knew they were passing legislation that if you didn't tick off donor then you would automatically assumed to be a donor. And, actually it is because of this change that made Mom and I started talking about it one day.

NS_004

I think it is having an impact. I think we've seen some positive numbers with respect to viable organs for transplant. It has been hard to measure cause and effect of anything with all the COVID and everything [illegible]

Supplement 1b Participant themes

Code	Quote
Barriers (to donation)	
-waiting	Once we got to Halifax, the people were good, but you were only allowed 3 in the hospital because of COVID so they gave us a family room. It was a room with no windows. It was like 10 x 10 with a couch. The worst part about it was that it took 3 days. So, that is a long period, three days, before they decided what they were going to take. Now I know they need to line up the others who need the organs but 3 days? The sitting around for three days waiting for them to make up their minds, which I guess is how it works but don’t leave

	everybody sitting there. I am going to change my donor card to no. I just can't put my family through that. (NS_017)<br><br>They had [pt name] in a negative pressure room and they mentioned something about that but anyways that's why he was in that room and I don't know it seems like an hour or so before they moved him to take him up for a CT scan and they sort of let us touch him for a second in the hallway as they were passing by to go for CT. And then from the CT it was hours, about three hours, before we saw him again in the ICU (NS_001A&B)<br><br>We told them that we can't wait forever. Like I can't wait for more than 3 days. We just couldn't wait any longer to get themselves organized. My husband stayed while the nursing staff prepped him for surgery, then we left. An older nurse told me that she'd take care of him like her grandchildren. She promised.(NS_001A&B)
-questionnaire	I went through the [profanity] roof, I jumped over the table at the woman who started asking me about [pt.'s name] and sexually transmitted diseases, I swear I could have [profanity] ripped off her face. I mean like he is [pt.'s age, pediatric] [profanity]; he hasn't been sexually abused. [profanity] off. Like gonorrhea. Like, [profanity], I lost it. (NS_001A&B)<br><br>It was terrible that they had to ask me if my dad ever had sex with other men, did I know if he was a homosexual, and did he have any diseases that I knew were sexually transmitted. Like, at the moment of losing your parent, those questions mad me feel so unsettled, like I had nothing to hide but I didn't want to think of those things either. (NS_015)
Transfer to tertiary hospital	They tried to get what they call Life Flight here which is the same as air ambulance. And they can land in Digby, right beside the hospital but they weren't flying because it was too windy. And they have a fixed wing which they can land close to Digby and it wasn't flying either so they said, 'ok we're going to have to go by road which is a two hour ride to Halifax. (NS_021)
Support	I'm not sure there would ever be enough support just because of the situation but I don't think we ever went without anything we needed. The nursing staff were beyond exemplary, just went out of their way to make us comfortable . They brought water for us. They did fingerprints and they took heartbeat recordings, and they like trimmed his hair and gave us each locks of his hair. They treated him with dignity and respect and that was huge to see how they were dealing with him. (NS_006)

	The main thing for us is we were treated like royalty. We couldn't have asked for better people to look after [name]. I mean that part was just wonderful as far as we were concerned. (NS_012)<br><br>The social worker followed up after we left the hospital. I talked with her about some of the experiences and some of the challenges the kids had afterwards because I know that she's been involved with multiple families and I was just trying to figure out what is normal grief and what's a red flag that they might need more help.<br><br>The staff in the hospital were so good to me and my Dad, I can never say enough good things. The day Dad passed the doctor came in and offered me his phone and asked me if I wanted to use it to play some music for my Father. And, of course, I played his group. That's how caring they were. Like, the doctor, gave me his phone for two hours. (NS_015)
Quality of care	The nurses were phenomenal. They were giving care as if he was still alive. Even when they were taking blood are doing whatever they were like, "here's a little poke [pt. name]. They treated hi like he was still a living child. (NS_001A&B)<br><br>We had a female ICU doctor and she was really nice and she ended up taking her mask off while she was talking to us because she was crying. She kneeled right down in front of us. (NS_001A&B)
COVID Context	We brought up donation and were surprised. It was like they were woah we're not there yet, but they also panicked because they weren't doing transplants, they shut that all down and the teams had been redistributed. We found out later if I'm remembered the details correctly, is that our request at that moment caused them to have some official conversations amongst themselves to pull together a team which had been on hold because of COVID, and they started doing some work as a group to pull together some surgeons and some stuff to make it happen. (NS_001A&B)<br><br>We had no restrictions even though it was COVID (NS_001A&B)<br><br>The donation was affected because of COVID something like they couldn't link up with Ontario and Quebec so they couldn't take part of

	his lungs, I’m not sure if it was because part of his lung was punctured. (NS_001A&B)<br><br>And with COVID it got a bit confusing because I think that communication could have been a little better when it came to how many people could be with [pt name] at a time. They gave us a family room so the three or four of us would sit in there and the other two would stay with [pt name]. We split up so somebody was always with her.(NS_003)<br><br>While in Turo we weren’t allowed to go in together and we weren’t allowed to get close because of COVID, when they transferred him to Halifax there was only one day that we weren’t allowed to go together, once they realized how bad it was there were no restrictions for the rest of the week. (NS_006)<br><br>Because of COVID it took another week to organize the donation.(NS_006)<br><br>Because of COVID only 2 people could be with him at a time (NS_012)
